# Cardiovascular Risk Factors and White Matter Hyperintensities: Difference in Susceptibility in South Asians Compared With Europeans

**DOI:** 10.1161/JAHA.118.010533

**Published:** 2018-10-31

**Authors:** Carole H. Sudre, Lorna Smith, David Atkinson, Nish Chaturvedi, Sébastien Ourselin, Frederik Barkhof, Alun D. Hughes, H. Rolf Jäger, M. Jorge Cardoso

**Affiliations:** ^1^ School of Biomedical Engineering and Imaging Sciences King's College London London United Kingdom; ^2^ Dementia Research Centre UCL Institute of Neurology London United Kingdom; ^3^ MRC Unit for Lifelong Health and Ageing at UCL Department of Population Science & Experimental Medicine UCL Institute of Cardiovascular Science London United Kingdom; ^4^ Centre for Medical Imaging UCL Division of Medicine London United Kingdom; ^5^ Department of Medical Physics and Biomedical Engineering University College London Malet Place Engineering Building London United Kingdom; ^6^ Department of Radiology and Nuclear Medicine Neuroscience Campus Amsterdam VU University Medical Center Amsterdam The Netherlands; ^7^ Lysholm Department of Neuroradiology The National Hospital for Neurology and Neurosurgery London United Kingdom; ^8^ Brain Repair and Rehabilitation UCL Institute of Neurology London United Kingdom

**Keywords:** aging, cerebral small vessel disease, ethnicity, magnetic resonance imaging, risk factor, white matter, Aging, Risk Factors, Magnetic Resonance Imaging (MRI), Race and Ethnicity

## Abstract

**Background:**

Cardiovascular risk factors vary between ethnicities but little is known about their differential effects on white matter hyperintensities (WMH), an indicator of brain aging and burden of cerebrovascular disease.

**Methods and Results:**

Brain magnetic resonance imaging scans from 213 people of South Asian and 256 of European ethnicity (total=469) were analyzed for global and regional WMH load. Associations with cardiovascular risk factors and a composite cardiovascular risk score (National Cholesterol Education Programme Adult Treatment Panel III) were compared by ethnicity, diabetes mellitus, smoking, and hypertension status. Distributional patterns of WMH were similar by ethnicity but the vulnerability to specific risk factors differed. Associations between WMH and age or National Cholesterol Education Programme Adult Treatment Panel III scores were stronger in South Asians compared with Europeans. For instance, a year of age led to an excess of 3.8% (confidence interval=[0.2, 7.6]; *P*=0.04) of WMH load in frontal regions in South Asians compared with Europeans. In the diabetic subgroup, South Asians had more WMH than Europeans (+63.3%, confidence interval=[14.1, 133.9]; *P*=0.007), particularly in the deeper regions (+102% confidence interval=[24, 329]; *P*=0.004). In the population as a whole, diabetes mellitus was not, or only weakly, related to an increase in WMH volume (12.4%, confidence interval=[−10.7, 41.3]; *P*=0.32), and diabetes mellitus duration was a positive predictor of frontal periventricular WMH load in Europeans but not in South Asians. In turn, diastolic blood pressure was positively associated with WMH volumes in South Asians but not in Europeans. Hypertension was not associated with WMH load (*P*=0.9).

**Conclusions:**

Distribution patterns of WMH are similar in South Asians and Europeans but older age and higher cardiovascular risk are associated with more WMH in South Asians.

**Clinical Perspective**

**What Is New?**

- White matter hyperintensities in the brain have a similar distribution pattern in South Asians and Europeans, but their association with cardiovascular risk factors differs.
- Diastolic blood pressure is strongly associated with white matter hyperintensities in the South Asian population, while the association with diabetes mellitus duration is stronger in Europeans.

**What Are the Clinical Implications?**

- Ethnicity should be considered for the assessment of brain vulnerability to cardiovascular risk factors and the prevention of cerebrovascular damage.

## Introduction

As part of the aging process, brain tissue damage accumulates and becomes visible with magnetic resonance imaging. Myelinated white matter axons are commonly affected by a compromised blood supply and/or dysfunction of the blood–brain barrier. With the degradation of the myelin sheath surrounding the axons, the relaxation time of the tissue is altered, resulting in a hyperintense signal on T2‐weighted and Fluid Attenuated Inversion Recovery magnetic resonance imaging sequences. White matter hyperintensities are the most common markers of age‐related cerebrovascular damage and neurovascular disease. They have been associated with various forms of cognitive decline^1^, ^2^, ^3^ and linked to the onset of dementia.^4^, ^5^

Given the commonly acknowledged vascular component in the pathophysiology of these brain tissue changes, it is not surprising that various cardiovascular risk factors have previously been associated with the presence of WMH including age, diabetes mellitus, and elevated blood pressure.^6^, ^7^, ^8^ Coronary heart disease,^9^ stroke,^10^ and type 2 diabetes mellitus^11^ are more common in South Asians than Europeans. The increased risk of cardiovascular disease in South Asians is not fully explained by known risk factors,^12^ and little is known about differences in association between risk factors and brain pathology in different ethnic groups.

In this study, we investigated cerebrovascular white matter damage in relation to brain aging in South Asian and European populations by investigating the pattern of WMH distribution, and the differences in associations between cardiovascular risk factors and neurovascular lesion burden.

## Material and Methods

The data that support the findings of this study are available from Dr Therese Tillin (t.tillin@ucl.ac.uk) upon reasonable request.

### Study Participants

**Figure 1:**
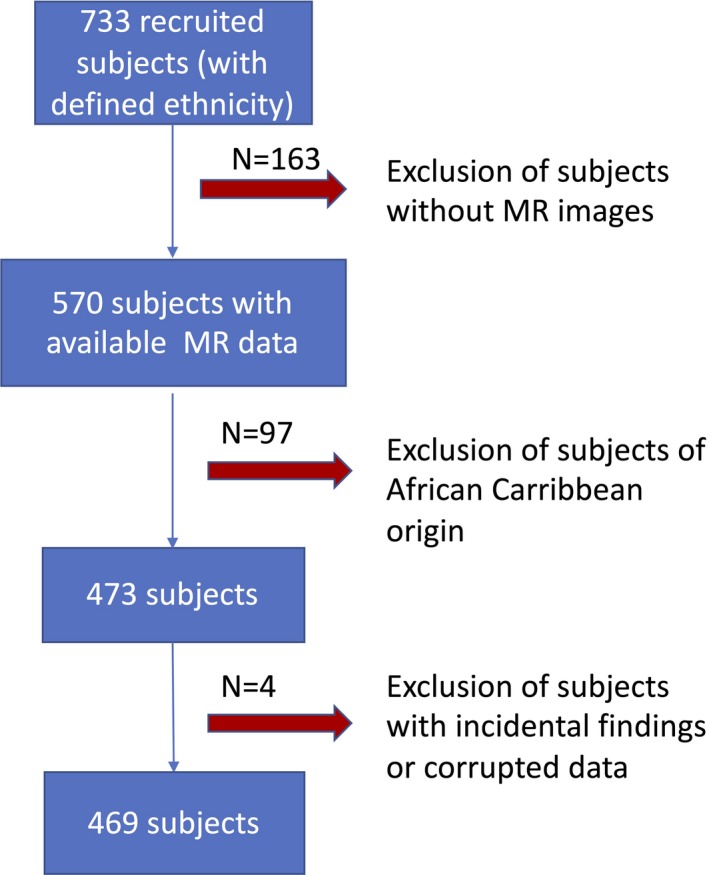
Flowchart of data selection. MR indicates magnetic resonance.

The SABRE (Southall and Brent Revisited) study^13^ was initiated in 1988 to 1991 with the aim of investigating the relationship between cardiometabolic risk factors and chronic disease in a multiethnic cohort of individuals. Men and women aged 40 to 69 years were recruited from the community in West and North West London, using primary care registration and workforce occupational logs as the sampling frame. Participants were only excluded from recruitment if they were too ill to travel to a local clinic, or could not provide written, informed consent. The 3 ethnicities represented in the SABRE cohort are European white, South Asian, and African Caribbean. Ethnicity was agreed on with the interviewer based on self‐report and parental country of birth. Following the definition established in the UK census of 2001, the South Asian ethnicity refers to individuals with Indian, Pakistani, or Bangladeshi ancestry. All South Asians and African Caribbeans were first‐generation migrants. For the third follow‐up visit on which this cross‐sectional work is based, index participants were invited along with their life partner. Visit 3 of the SABRE study received ethical approval from the National Research Ethics Service Committee, London—Fulham (14/LO/0108) and all participants gave written informed consent. Data presented here were acquired on a subset of participants who attended clinic between September 2014 and December 2016. Because of the limited number of individuals of African Caribbean origin in this sample, we limited our analysis to a comparison of South Asian and European individuals (n=469; 213 South Asians, 256 Europeans) after exclusion of 1 subject with type 1 diabetes mellitus, 1 subject with a meningioma, and 2 subjects for whom imaging data were corrupted. The data selection process is illustrated in Figure 1.

**Figure 2:**
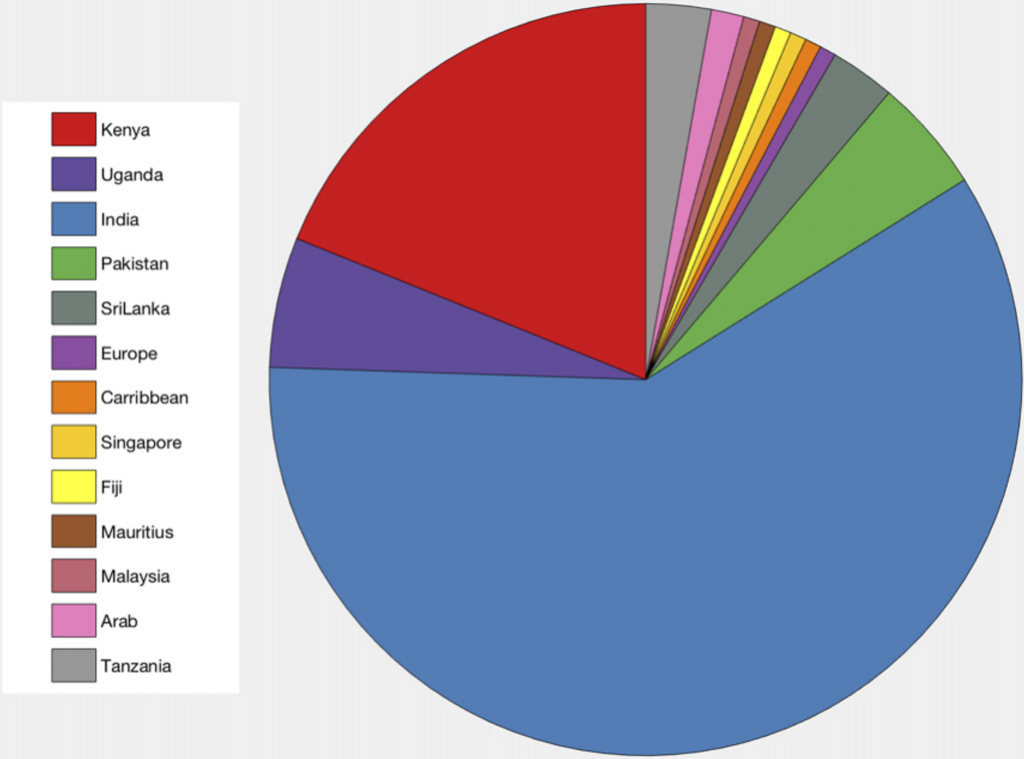
Repartition of previous country of residence before migration in South Asian population.

**Figure 3:**
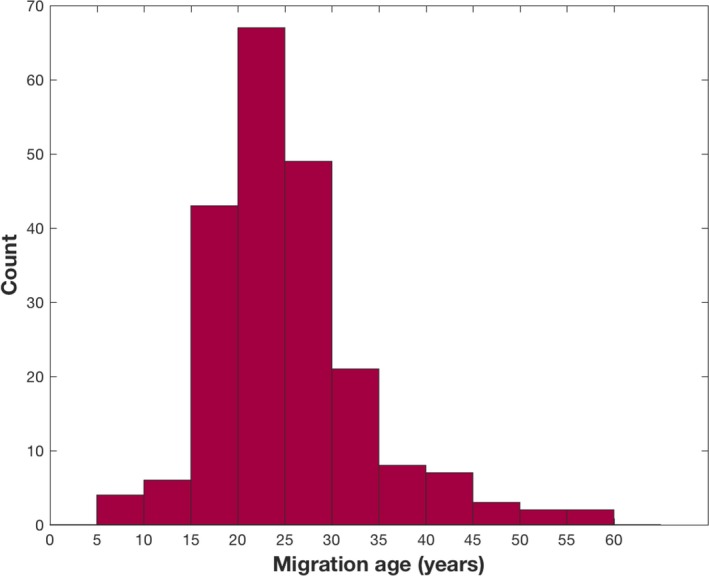
Histogram of age at migration for the South Asian population.

In order to further characterize the South Asian population, Figures 2 and 3 display information on their previous country of residency and their age at migration.

### Clinical Assessment

Seated clinic blood pressure (BP) was measured according to European Society of Hypertension/European Society of Cardiology 2013 guidelines. In short, BP was measured in both arms after 5 minutes of rest using an Omron MIT Elite Plus blood pressure monitor with an appropriate cuff size. Clinic BP was calculated as the average of the final 2 measurements of a minimum of 3 measures in the left arm unless the difference between arms was >10 mm Hg, in which case the arm with the higher BP was used. Blood was drawn and results of total cholesterol and high‐density lipoprotein cholesterol were used for cardiovascular risk score assessment. Smoking habits, hypertension diagnosis, diabetes mellitus status and duration were obtained from questionnaires completed before the visit. In order to account for linguistic requirements, questionnaires were also available in the native language of the participants. The clinical measurements were combined in a cardiovascular risk score (10‐year risk of coronary heart disease) derived from the National Cholesterol Education Programme Adult Treatment Panel III (NCEP ATP III) guidelines.^14^ This score is built as the sum of risk points attributed per sex and age range for age, measures of total cholesterol, high‐density lipoprotein cholesterol, systolic BP including correction for treatment, and smoking status. The details of the building of the score can be found in the guidelines.^14^

### Magnetic Resonance Imaging Acquisition

Magnetic resonance imaging images were acquired at a single site on a 3T Philips Achieva scanner on the same day as the clinical examination. For this study, 2 of the acquired structural sequences were used:

1. Three‐dimensional sagittal T1‐weighted multishot, inversion‐prepared gradient echo: repetition time 6.9 ms; echo time 3.1 ms; voxel size 1.0×1.0×1.0 mm^3^;
2. Three‐dimensional sagittal T2‐weighted Fluid Attenuated Inversion Recovery: repetition time 4800 ms; inversion time 1650 ms; echo time 125 ms; voxel size 1.0×1.0×1.0 mm^3^.

### Image Postprocessing

**Figure 4:**
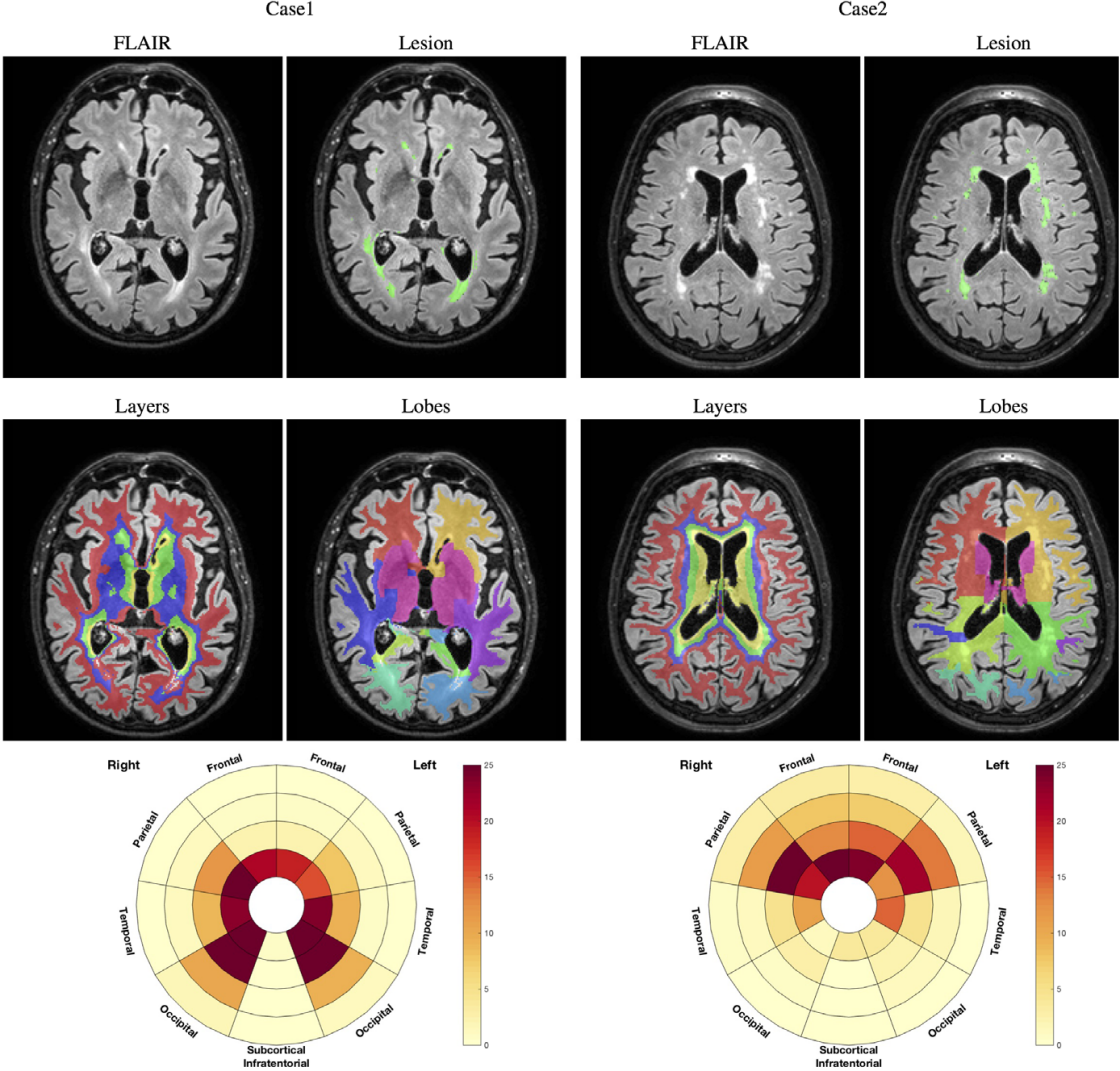
Example of 2 cases with original FLAIR image and lesion segmentation (first row), layers and lobar separation (second row), and resulting bullseyes plot. The color indicates the percentage of a given region to be occupied by WMH. The angular segment represents the different lobes while concentric layers indicate distance layers from the ventricular surface, distance increasing with the radius. Example of 2 cases with original FLAIR image and lesion segmentation (first row), layers and lobar separation (second row), and resulting bullseyes plot. The color indicates the percentage of a given region to be occupied by WMH. The angular segment represents the different lobes while concentric layers indicate distance layers from the ventricular surface, with distance increasing with the radius. FLAIR indicates Fluid Attenuated Inversion Recovery; WMH, white matter hyperintensities.

Using T1‐weighted images, the cortical gray matter was automatically parcellated into lobes using Geodesic Information Flows, a label fusion algorithm.^15^ This algorithm was also used to skull‐strip the images as well as provide subject‐specific tissue probabilistic atlases. These probabilistic atlases and brain masks were used as inputs to the BaMoS algorithm,^16^ in order to automatically segment WMH. This segmentation algorithm models lesions as a Gaussian Mixture Model under multivariate data (T1‐w, Fluid Attenuated Inversion Recovery) and automatically determines the number of Gaussian components required to jointly model healthy tissues and abnormal signals. After convergence, the optimized model is used to produce a probabilistic lesion map that is integrated to produce lesion volume measurements. All lesion segmentations passed quality control by visual assessment. In order to spatially localize WMH, a subject‐specific coordinate frame was used to divide the WM and deep gray matter by cortical lobe, and distance from the ventricular surface. Cortical lobes were defined by solving the Laplace equation between the ventricular surface and aggregate Geodesic Information Flows lobar regions, dividing the brain into 5 lobes and 4 radial layers. Spatial localization was illustrated in a “bullseye's representation” of WMH as described previously^17^ and illustrated in Figure 4 for 2 cases. Finally, WMH volumes were estimated per region, providing regional summary measures and allowing the 3‐dimensional WMH distribution to be displayed as a 2‐dimensional plot (Figure 4).

### Statistical Analysis

Continuous descriptive sample data are presented as means (SD) or median (interquartile range) for skewed data; other results are shown as means (95% confidence intervals [CI]). Categorical data are presented as frequencies (%). Simple comparisons were made using a Student *t* test for continuous data (after transformation if necessary) and a χ^2^ test for categorical data. In order to account for the skewness of WMH volume data, a Generalized Linear Model with a gamma distribution and a log link was used. As a result, the obtained β coefficients from the regression are interpreted as a multiplicative factor and are presented as a percent increase per unit increase of predictor. Results are presented with their 95% CI and associated *P* value. Total intracranial volume (TIV), age, sex, and ethnicity were included in all models as potential confounders. When investigating age effect across ethnicities, an interaction term between age and ethnicity was included in the model.

Investigation of the relationship between cerebrovascular risk factors and WMH volumes were conducted, stratified for each ethnic group, adjusting in each case for age, sex, and TIV. Associations with diabetes mellitus were studied in 2 ways: first by including diabetes mellitus as a binary categorical variable, secondly by including diabetes mellitus duration as a continuous variable for people with diabetes mellitus. Similarly, associations with BP were assessed using the diagnosis of hypertension as a binary variable, and by analyzing associations with BP as a continuous variable in the hypertensive group correcting for age, sex, and TIV. In order to account for the effect of antihypertensive medication in this analysis, 10 (resp. 5) mmHg was added to the measured value when the subject was receiving antihypertensive treatment as discussed.^18^, ^19^ Because of the limited number of current smokers (8 Europeans, 2 South Asians), smoking was considered as a binary variable by pooling the current smoking and ever‐smoked categories; associations with duration of smoking were also analyzed in the smoking group. All analyses were performed using Stata v.14; *P*<0.05 was considered statistically significant.

## Results

Demographic and volumetric data are presented in Table. On average, South Asians were 1.5 years younger than Europeans, had a higher prevalence of diabetes mellitus and hypertension and lower concentrations of total and high‐density lipoprotein cholesterol. Diabetes mellitus duration was slightly longer for South Asians compared with Europeans and showed a larger variance. No major difference was observed across ethnicities in terms of NCEP ATP III score, although the variance was higher in Europeans.

**Table 1:** Demographic, Health, and WMH Characteristics Stratified by Ethnicity and for the Whole Sample

	European (N=256)	South Asian (N=213)	Total (N=469)	*P* Value
Female, N (%)	90 (35.2)	88 (41.3)	178 (38)	0.17
Diabetes mellitus diagnosed, N (%F)	38 (23.7)	66 (28.8)	104 (26.9)	<0.0005
Hypertension diagnosed, N (%F)	123 (29.3)	146 (32.2)	184 (30.9)	<0.0005
Hypertension treated, N (%F)	113 (27.4)	144 (33.3)	182 (30.7)	<0.0005
Ever smoked, N (%F)	151 (28.4)	33 (3.0)	184 (23.9)	<0.0005
Age, y	72.2 (5.9)	70.7 (5.9)	71.5 (5.9)	0.007
Systolic blood pressure, mm Hg	129.8 (17.4)	131.3 (17.4)	130.5 (17.4)	0.37
Diastolic blood pressure, mm Hg	79.3 (8.1)	78.5 (7.7)	78.9 (7.9)	0.30
Heart rate, beats/min	65.1 (10.9)	63.4 (10.8)	64.4 (10.9)	0.10
Diabetes mellitus duration, y	12.1 (6.2)	15.2 (8.8)	14.1 (8.1)	0.07
Smoking duration, y	24.5 (16.5)	23.1 (18.0)	24.2 (16.7)	0.67
Total cholesterol, mg/dL	187.3 (40.9)	175.7 (40.1)	182 (40.9)	0.002
High‐density lipoprotein cholesterol, mg/dL	62.4 (18.3)	57.5 (15.1)	60.2 (17.1)	0.002
ATP III score	15.2 (2.9)	14.9 (2.6)	15.0 (2.8)	0.31
Total intracranial volume, mL	1381.3 (125.6)	1246.7 (116.9)	1320.7 (138.4)	<0.0005
WMH load, mm^3^				
Total	3242.7 [1677.6; 9257.6]	2471 [1328.2; 7236.6]	2971.4 [1551.1; 7692.1]	0.87
Frontal	1779.0 [839.7; 4477.4]	1378.6 [627.2; 3835.2]	1582.4 [753.5; 4101.7]	0.90
Parietal	432.6 [160.4; 1678.9]	254.3 [113.1; 1071]	340.5 [137.1; 1334.7]	0.75
Occipital	384.8 [209.3; 719.9]	338.5 [188.3; 694.6]	364.1 [201; 708.5]	0.90
Temporal	271.4 [104; 742.9]	187.7 [82.3; 528.3]	214.3 [93.3; 630.8]	0.65
Subcortical/infratentorial	140.0 [67.6; 228.6]	142.5 [75.9; 272.2]	141.4 [71.9; 258.5]	0.28
Periventricular	1422.6 [756; 2922.2]	1079.3 [530.1; 2263.3]	1239.8 [659.5; 2685.5]	0.86
Median	992.5 [536.4; 3748.3]	788.2 [370.7; 2769]	909.0 [458; 3340.2]	0.998
Juxtacortical	570.0 [211.3; 1500.5]	530.2 [219.7; 1221.1]	547.0 [217.6; 1319.4]	0.60

*P* values for the comparison between Europeans and South Asians are indicated in the last column. Comparison for the lesion load is performed after correction for age, sex, and total intracranial volume. Counts are presented with (%F) as percentage of females, WMH volumes in the format median [first quartile; third quartile], and other measures with the format mean (SD). ATP III indicates National Cholesterol Education Programme Adult Treatment Panel III; WMH, white matter hyperintensities.

**Figure 5:**
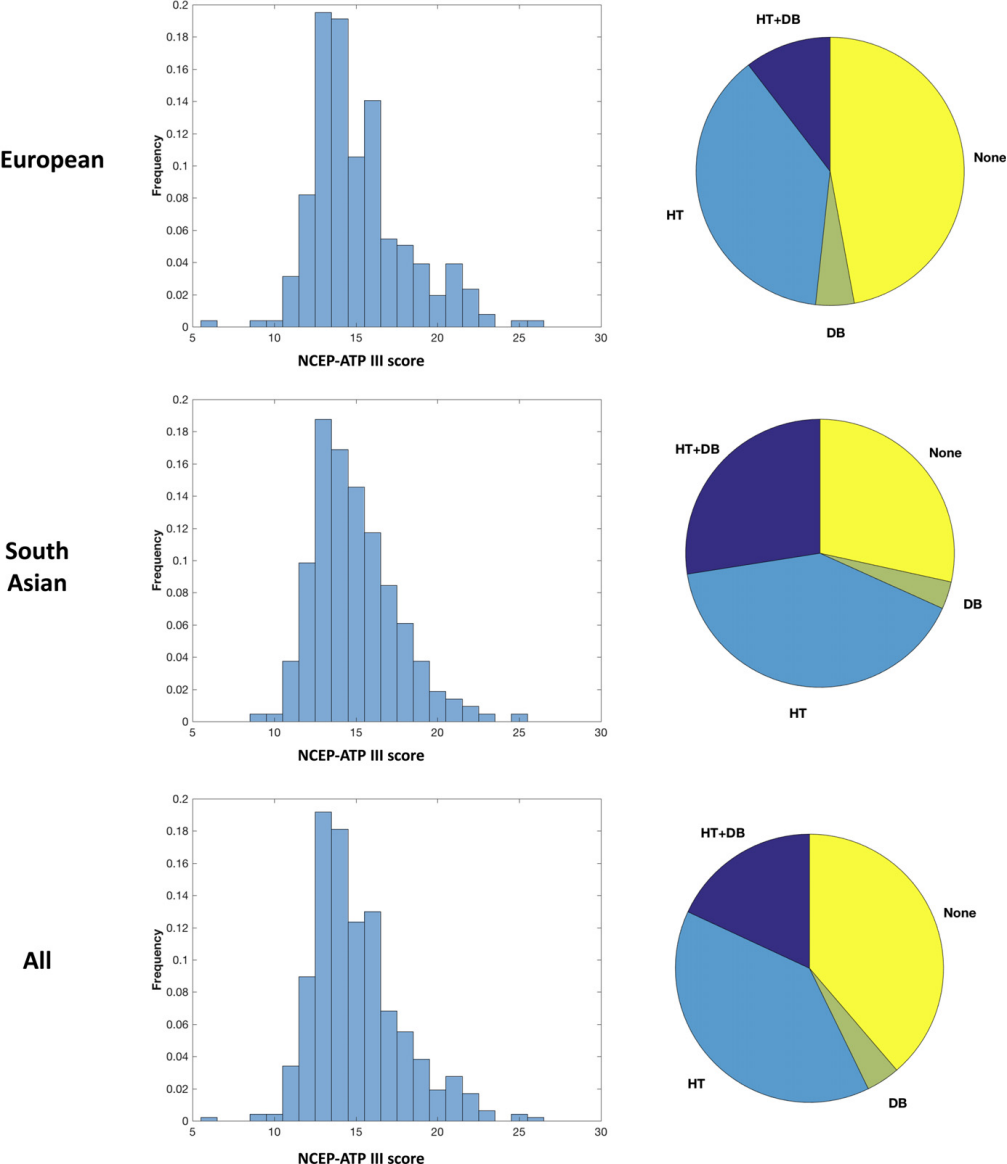
Distribution of NCEP ATP III scores (left) for European (top) South Asian (middle), and overall (bottom), and proportions with key disease burden (right). DB indicates diabetes mellitus; HT, hypertension; NCEP ATP III, National Cholesterol Education Programme Adult Treatment Panel III.

Figure 5 shows the NCEP ATP III scores across ethnicities as well as the prevalence of hypertension and diabetes mellitus.

### WMH Analysis

TIV as surrogate of head size was positively associated with lesion load, 1 mL of excess in TIV being associated with 0.015% of additional WMH load (CI=[0.008, 0.02]; *P*<0.0005) and was thus consistently corrected for in all analyses. Although there was no sex difference for overall lesion load, females had a higher load in the frontal (excess of 28.6% CI [3.3, 48.4]; *P*=0.033) and periventricular regions (excess of 23.8% CI [5.8, 37.9]; *P*=0.011), when compared with males. No difference was observed between ethnicities with regard to WMH burden (Table), both overall and locally after correction for age, sex, and TIV.

**Figure 6:**
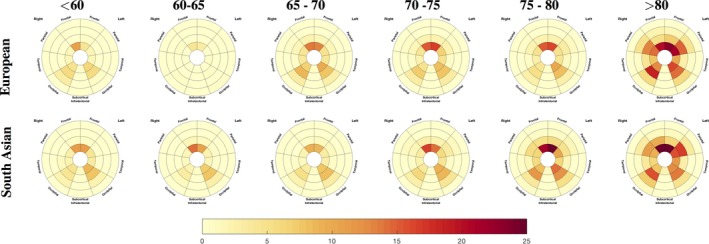
Median load of WMH by location for each 5‐year age interval for the 2 ethnic groups. Red colors correspond to a higher occupancy ratio of the associated region. Radial distance layers correspond to the distance from the ventricular system. WMH indicates white matter hyperintensities.

**Figure 7:**
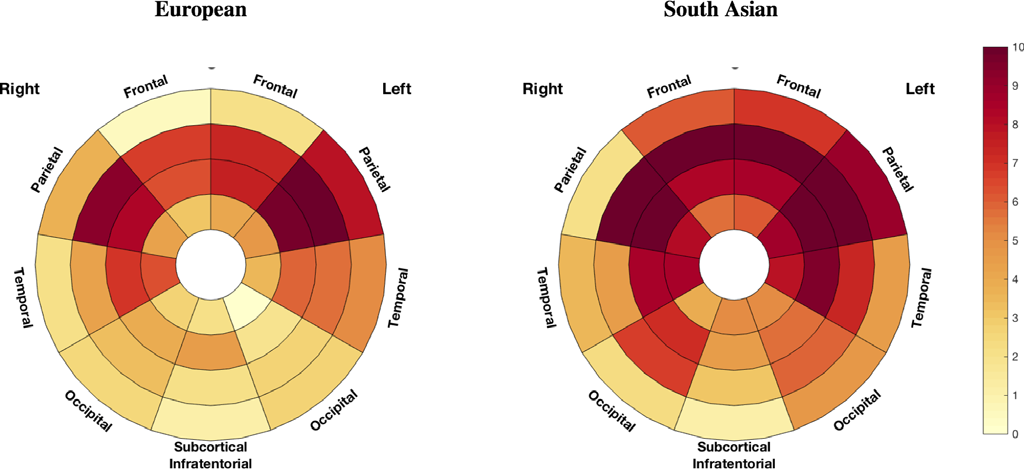
Differences in age effect by brain location and ethnicity. The % of the difference in WMH load associated with 1 year of age is plotted in the bullseye. Darker red reflects a stronger increase in WMH for each year of increase in age. WMH indicates white matter hyperintensities.

WMH load and distribution correlated with age. The pattern of WMH distribution associated with age showed strong similarities between the 2 ethnic groups (Figure 6). In younger people, WMH were observed predominantly in the frontal periventricular regions, while extension into the parietal and occipital lobes and more peripheral regions was evident in older people. Despite following a similar geographical pattern with age, WMH load accumulation from 1 age stratum to the other was greater in South Asians (Figure 6), implying that age has a more detrimental effect on South Asians than Europeans. As might be expected, this interaction between age and ethnicity was most prominent in regions with the greatest WMH load, ie, the frontal (+3.8% per year of age in South Asian compared with European CI=[0.2, 7.6]; *P*=0.041), occipital (3.3% CI=[0.4, 6.4]; *P*=0.036) and most periventricular regions (3.0% CI=[0.5, 5.5]; *P*=0.018) and slightly weaker for total lesion load (3.3% CI=[−0.3, 6.9]; *P*=0.07). Figure 7 presents the per‐year of age % increase in lesion load between ethnicities.

### Link Between Cardiovascular Risk Factors and WMH

#### NCEP ATP III score

The NCEP ATP III score was positively associated with overall lesion load (8.8% CI=[4.4, 13.4] increase per 1‐unit increase in score; *P*<0.0005). This relationship was observed in all lobes and layers except for the occipital lobe. With respect to ethnicity, associations with WMH were stronger in the South Asians except for the basal ganglia. For the Europeans, significant associations were only observed in the basal ganglia, the frontal lobe, and the first 2 most periventricular layers.

**Figure 8:**
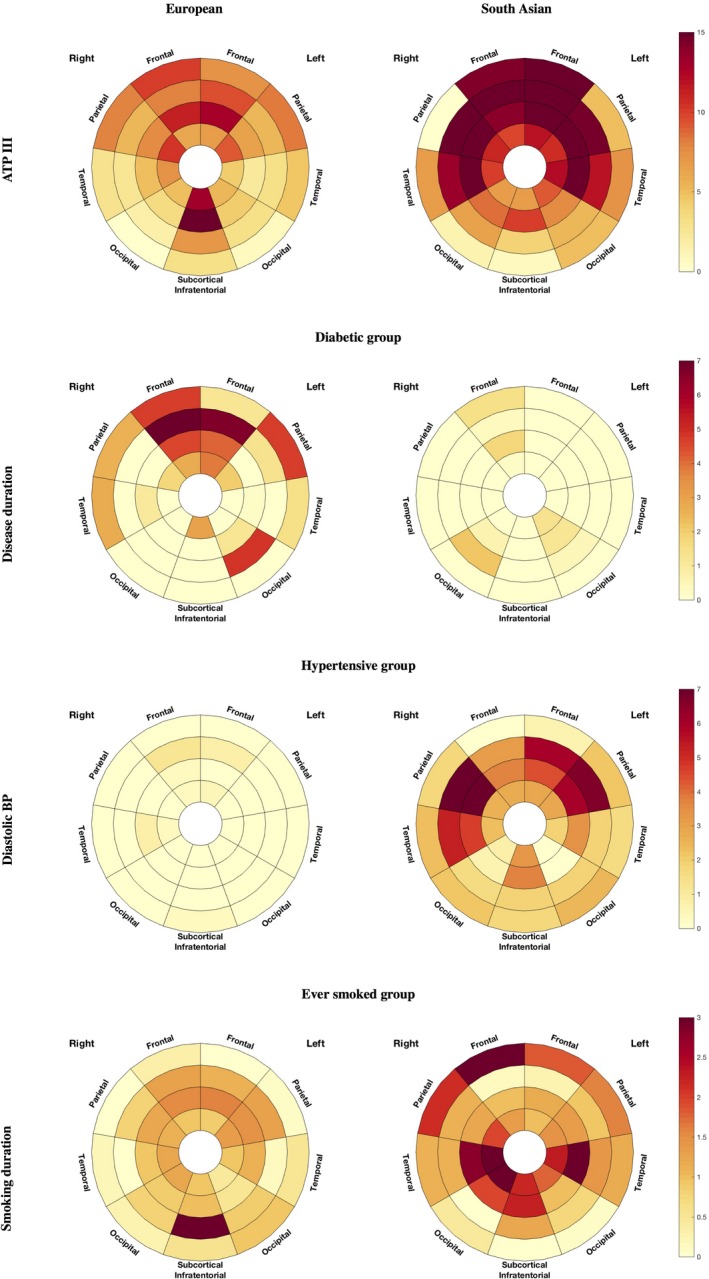
Localized association of white matter hyperintensities (WMH) with ATP III score (top row), disease duration for the diabetic group (middle row), and diastolic blood pressure for the hypertensive group (bottom row) for the European (left) and South Asian (right) population. Darker regions correspond to a higher percentage of increase of WMH per increment of the corresponding independent variable (ATP III point score, years of diabetes mellitus disease duration, blood pressure point, smoking duration). ATP III indicates National Cholesterol Education Programme Adult Treatment Panel III; WMH, white matter hyperintensities.

Figure 8 presents on the first row the percentage of additional WMH load associated with 1 more point of NCEP ATPIII score for the 2 ethnicities in each of the 36 predefined regions.

#### Diabetes mellitus

**Figure 9:**
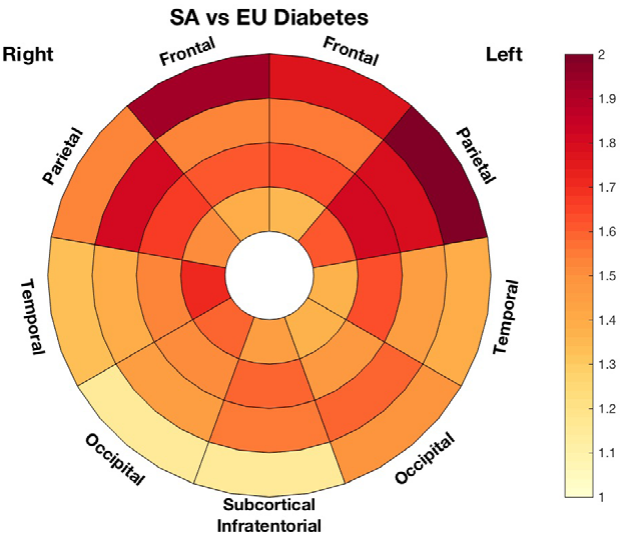
Multiplicative factor of differences in white matter hyperintensities load between diabetic South Asian (SA) and European (EU) groups.

Diabetes mellitus was not convincingly associated with higher lesion load, either when considering the population as a whole (12.4% CI=[−10.7, 41.3]; *P*=0.32) or in South Asian (20.7% CI=[−11.4, 64.3]; *P*=0.2) or European (7.1% CI=[−24.8, 50.5]; *P*=0.7) subgroups separately. However, South Asians diagnosed with diabetes mellitus had a higher overall WMH load than Europeans, with a 63.3% (CI=[14.1, 133.9]; *P*=0.007) increase in WMH volume. Similar associations were found for each lobe and layer separately, except for the occipital lobe and the most periventricular region. The difference across ethnicities was strongest in the most juxtacortical regions with an excess of 102% (CI=[24, 329]; *P*=0.004) in the South Asians. The regional plot highlighting the multiplicative factor of difference in lesion load between diabetic South Asian and European is presented in Figure 9.

Figure 8 (second row) displays the percentage of additional WMH volume associated with 1 more year of diabetes mellitus in the European and South Asian population for all 36 regions.

When investigating separately the 2 ethnic groups, duration of diabetes mellitus was only significantly associated with lesion load in the frontal periventricular regions for the European population (left: 3.9% CI=[0.0, 7.3]; *P*=0.044; right: 2.7% CI=[0.2, 5.3]; *P*=0.035 more volume per 1 year of diabetes mellitus). The whole frontal lobe and the most periventricular layer were of similar magnitude (4.2% CI=[−0.3, 8.8]; *P*=0.06 and 2.66% CI=[−0.5, 5.7]; *P*=0.10), albeit without achieving statistical significance. There seemed, however, to be a gradient in the magnitude of the effect towards the deeper regions. This pattern was not observed in the South Asian population, for which there was no association between lesion load and disease duration (frontal periventricular left: −0.5% CI=[−3.2, 2.3]/ frontal periventricular right: 0.2% CI=[−1.8, 2.3] frontal: 0.1% CI=[−3.4, 3.8]/ periventricular: −0.3% CI=[−2.7, 2.2]).

#### BP and hypertension

**Figure 10:**
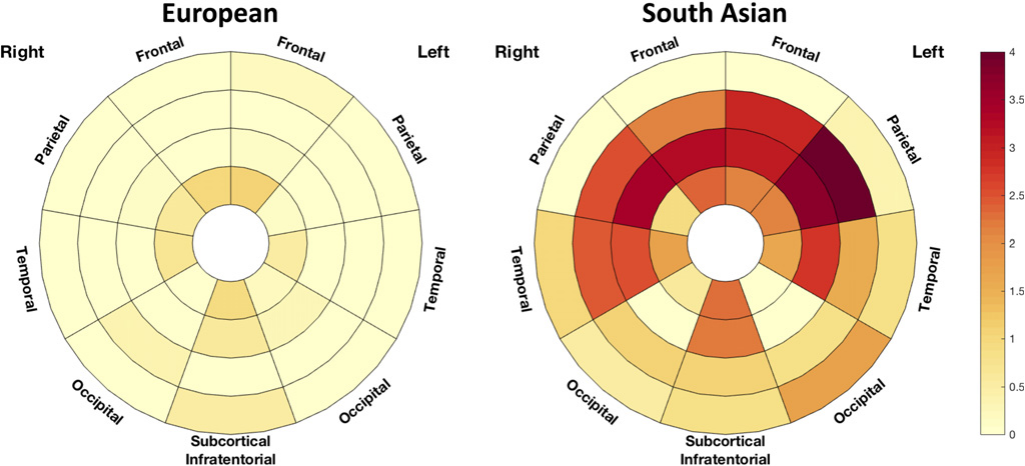
Localized association of white matter hyperintensities with diastolic blood pressure on the whole population across ethnicities.

Hypertension was not associated with WMH load, either in the sample as a whole, or in ethnic subgroups. Contrary to our observations in diabetes mellitus, in analyses restricted to hypertensives, there was no difference in WMH load (globally or locally) between ethnicities (*P*=0.97). In South Asian hypertensive patients, there was, however, a positive association between diastolic BP and WMH in all lobes except the occipital region, and all layers except the most juxtacortical one (Figure 8, third row). In this subgroup, the overall lesion load increased by 2.9% (CI=[0.5, 5.2]; *P*=0.015) per 1 mm Hg higher diastolic BP. The effect was notably strong in the parietal lobe with an increase of 5.0% (CI=[2.2, 8.0]; *P*=0.0003) per mm Hg higher diastolic BP. A similar, but less prominent (overall 1.5% CI=[−0.2, 3.3]; *P*=0.09/parietal 1.9% CI=[−0.2, 4.1]; *P*=0.08), pattern of relationship was observed for the whole South Asian subgroup including both hypertensive and nonhypertensive participants. The plots of the relationship between diastolic BP and lesion load for the 2 whole populations are shown in Figure 10.

There was no association with diastolic BP in hypertensive Europeans (−0.05% CI=[−1.5, 1.4]; *P*=0.9). Neither ethnic group showed any associations between WMH and systolic BP (data not shown).

#### Smoking

A weak or null association was observed between smoking as a binary variable (never/ever) and higher periventricular WMH volume (15.3% CI=[−2.4, 36.2]; *P*=0.093), and there was no difference in WMH load between South Asians and Europeans who were current/ever smokers (South Asian +5% CI=[−33.2, 65.9]; *P*=0.819). When considering individuals who had ever regularly smoked, duration of smoking was associated with a higher lesion load both overall (1.1% excess per smoking year CI=[0.2, 2.0]; *P*=0.015) and for all lobar regions, notably the subcortical regions (1.4% excess per smoking year CI=[0.5, 2.2]; *P*=0.002), the periventricular (1.1% [0.4, 1.8]; *P*=0.003), and the midlayers (1.5% CI=[0.3, 2.6]; *P*=0.007). The distribution pattern of WMH associated with smoking differed across ethnicities, being more central for Europeans and stronger in the juxtacortical region in South Asians (1 year of smoking was found to be associated with an excess of 1.9% of WMH (CI=[0.0, 3.9]; *P*=0.05 in this region). The fourth row of Figure 8 displays the comparison in the association between years of smoking and WMH load across ethnicities.

## Discussion

Both in Europeans and South Asians, the rate of increase in volume of WMH was associated with age; the increase of WMH with age showed a fairly symmetrical pattern, spreading from the ventricles towards the juxtacortical region. There were, however, clear differences in associations between the percentage increase of WMH and risk factors between the 2 ethnic groups. The association between age and incremental WMH volume was stronger in South Asians than Europeans, with an excess increase of 2% of lesion load per year of age, which was most evident in the medial layers. This difference may not strictly reflect the effect of 1 additional year, but the overall effect of aging in the differential overall health context goes across ethnicities and thus encompasses the variations in other cardiovascular risk factor prevalence. ATP III cardiovascular risk scores were positively associated with WMH load in both ethnic groups, but this relationship was more pronounced in South Asians. South Asian people had a higher prevalence of diabetes mellitus and hypertension than Europeans, as has been previously observed.^9^, ^12^ Among people with diabetes mellitus, South Asians had a higher WMH load in most of the lobes and layers despite being on average younger than Europeans with diabetes mellitus. There was no association between hypertension or systolic BP and WMH load in either ethnic group, but higher diastolic BP was associated with greater WMH load in South Asians. These findings indicate that some key cardiovascular risk factors are associated with higher vulnerability for small vessel cerebrovascular disease in South Asian people, as has been previously reported for stroke.^12^

The association between WMH volume and diabetes mellitus duration was slightly stronger in Europeans. A likely explanation for this is the lower diabetes mellitus prevalence in Europeans, together with a greater variation in disease duration in Europeans. The longer duration of diabetes mellitus in South Asians, combined with the smaller variance and the higher overall load of WMH, may result in a “ceiling effect” obscuring relationships between WMH load and diabetes mellitus duration. Interestingly, Bryan et al^20^ showed a link between gray matter atrophy and diabetes mellitus duration but not with WMH volumes, although the studied population was much younger than that in SABRE.

The stronger relationship between diastolic BP and WMH volume in South Asian is in line with a recent study reporting stronger associations between diastolic BP and risk of stroke in this ethnic group.^21^

Although smoking as a binary variable was unconvincingly related to WMH burden, duration of smoking was positively associated with the WMH load. Interestingly, the pattern of impact of smoking duration was more peripheral for the South Asian than the European population. These findings, in relation to the differential effect of age, are of further interest when reflecting on the findings of Kim et al, who found an age dependence to the locational association between smoking and WMH.^22^

When comparing the effect of diabetes mellitus and diastolic BP on regional distributions of WMH, we observed more periventricular and parietal distribution of lesions in association with elevated diastolic BP, whereas diabetes mellitus was associated with more lesions in the juxtacortical areas. The association between periventricular lesion and diastolic BP is consistent with the findings of van Dijk et al,^8^ who reported a relationship between changes in diastolic BP over time and periventricular lesion load. The suggestion of a link between diabetes mellitus and WMH in the juxtacortical areas is consistent with recent findings of de Bresser et al,^23^ who found a higher prevalence of punctate juxtacortical lesions in subjects with diabetes mellitus. These findings warrant further investigations into the pathophysiological mechanisms by which various cardiovascular risk factors may influence the distributional burden of WMH.

The main strengths of our study lie in the novel automated analysis of WMH with detailed regional descriptors, the use of a well‐characterized cohort, and a standardized imaging protocol using volumetric image acquisitions at high field strength. The cross‐sectional nature of this study is a limitation since only associations can be studied, and further investigations on longitudinal data might help to ascertain causal relationships and shed light on the mechanisms linking cardiovascular risk factors to WMH and how they are modulated by ethnicity.

In addition, multiple statistical tests were performed in this study, and this naturally increases the risk of false discovery; thus, the results should be viewed with caution.

Another limitation is that some information, such as the age at diabetes mellitus diagnosis or smoking habit, is based on questionnaires and recall, which are prone to inaccuracies and misreporting. However, conditions requiring ongoing medical management, such as diabetes mellitus, have been reported to demonstrate good agreement between self‐report and medical records^24^; Pastorino et al have also recently shown that self‐recall of age of diagnosis of diabetes mellitus is highly accurate.^25^ In the future, ascertainment of disease status may be improved by linkage to electronic health record systems.^26^

It is also possible that differences in associations across ethnicities are affected by ethnic differences in the access to and use of healthcare systems, especially with respect the diagnosis and control of cardiovascular conditions. It has, however, recently been shown that there is negligible differential access between South Asians and white Europeans to the healthcare system for the diagnosis and monitoring of hypertension and diabetes mellitus in the United Kingdom.^27^ Another related issue is that South Asian participants were first‐generation migrants from countries in which healthcare systems may differ greatly. However, in almost all cases, migration took place well in advance of diagnosis of diabetes mellitus or hypertension (the average age at migration was 25.6 years, while the age of diagnosis of diabetes mellitus was 56.2); this is likely to limit this potential source of bias. Explicitly, only 1 subject was diagnosed with diabetes mellitus before migrating to England. Several avenues of further investigation are now open after this initial cross‐sectional investigation of differences in WMH pattern across ethnicities. Habits and lifestyle notably related to diet and physical activity may be relevant to understanding differential associations. On the imaging side, since vascular brain damage is not restricted to WMH, other imaging markers such as lacunes, cerebral microbleeds, and enlarged perivascular spaces that are associated with cardiovascular risk factors should also be investigated. Furthermore, the relationship between gray matter atrophy and WMH warrants more detailed investigation. Further studies of ethnic groups would contribute to building a clearer picture of the differential vulnerabilities and patterns of damage. A better understanding of the complex interactions between different cardiovascular risk factors and brain health has to be a priority for health systems catering to an increasingly old and diverse population, especially given the socioeconomic impact of cerebrovascular disease. This may ultimately result in a more tailored approach to risk factor management and lifestyle recommendations in different ethnic groups.

## Sources of Funding

Sudre is funded by Alzheimer's Society (AS‐JF‐17‐011). Ourselin receives funding from the Engineering and Physical Sciences Research Council (EPSRC) (EP/H046410/1, EP/J020990/1, EP/K005278), the Medical Research Council (MRC) (MR/J01107X/1), the EU‐FP7 project VPH‐DARE@IT (FP7‐ICT‐2011‐9‐601055). Cardoso receives funding from EPSRC (EP/H046410/1). Barkhof receives funding from the BRC. The Dementia Research Centre is supported by Alzheimer's Research UK, Brain Research Trust, and The Wolfson Foundation. The SABRE study was funded at baseline by the Medical Research Council, Diabetes UK, and the British Heart Foundation. At follow‐up, the study was funded by the Wellcome Trust (067100, 37055891 and 086676/7/08/Z), the British Heart Foundation (PG/06/145, PG/08/103/26133, PG/12/29/29497 and CS/13/1/30327) and Diabetes UK (13/0004774). Chaturvedi and Hughes receive support from the National Institute for Health Research University College London Hospitals Biomedical Research Centre, and work in a unit that receives support from the UK Medical Research Council (Programme Code MC_UU_12019/1). The SABRE study team also acknowledges the support of the National Institute of Health Research Clinical Research Network (NIHR CRN).

## Disclosures

None.
